# Galectin-3 Inhibition Ameliorates Streptozotocin-Induced Diabetic Cardiomyopathy in Mice

**DOI:** 10.3389/fcvm.2022.868372

**Published:** 2022-04-26

**Authors:** Ning Zhu, Liuyan Zhu, Bingwu Huang, Wenjun Xiang, Xuyong Zhao

**Affiliations:** ^1^Department of Cardiology, The Third Affiliated Hospital of Shanghai University, The Wenzhou Third Clinical Institute Affiliated to Wenzhou Medical University, Wenzhou People's Hospital, Wenzhou, China; ^2^Department of General Practice, The Third Affiliated Hospital of Shanghai University, The Wenzhou Third Clinical Institute Affiliated to Wenzhou Medical University, Wenzhou People's Hospital, Wenzhou, China; ^3^Department of Anesthesiology and Perioperative Medicine, The Second Affiliated Hospital and Yuying Children's Hospital of Wenzhou Medical University, Wenzhou, China; ^4^Department of Pathology, The Third Affiliated Hospital of Shanghai University, The Wenzhou Third Clinical Institute Affiliated to Wenzhou Medical University, Wenzhou People's Hospital, Wenzhou, China

**Keywords:** Galectin-3, diabetic cardiomyopathy, inflammation, macrophage, fibrosis

## Abstract

**Objective:**

Diabetic cardiomyopathy (DCM), characterized by cardiomyopathy with the absence of coronary artery disease, hypertension, and valvular heart disease in patients with diabetes, significantly increases the risk of heart failure. Galectin-3 (Gal-3) has been shown to regulate cardiac inflammation and fibrosis, but its role in DCM remains unclear. This study aimed to determine whether Gal-3 inhibition attenuates DCM and NF-κB p65 activation.

**Methods:**

Diabetic cardiomyopathy (DCM) was established by intraperitoneal (IP) injection of streptozotocin for 5 consecutive days in mice. Myocardial injury markers, such as creatine kinase isoenzyme (CK-BM) and lactate dehydrogenase, were detected using ELISA. We used non-invasive transthoracic echocardiography to examine cardiac structure and function. Histological staining was used to explore myocardial morphology and fibrosis. Profibrotic markers and inflammatory cytokines were detected by ELISA and real-time PCR *in vivo*. The terminal deoxyribonucleotide transferasemediated dUTP nick end-labeling (TUNEL) and immunofluorescence assays were conducted to examine myocardial apoptosis and oxidative stress. Inflammatory cytokines induced by high glucose (HG) were also found in RAW264.7 macrophages. The underlying molecular mechanisms were determined using immunofluorescence and Western blotting analyses.

**Results:**

The Gal-3 knockdown was observed to ameliorate myocardial apoptosis, oxidative stress, inflammatory cytokines release, macrophage infiltration, and fibrosis, thus, decreasing cardiac dysfunction in DCM mice. In addition, the silence of Gal-3 could suppress macrophage infiltration and inflammatory cytokine release induced by HG. Finally, a Gal-3/NF-κB p65 regulatory network was clarified in the pathogenesis of DCM.

**Conclusion:**

The Gal-3 may promote myocardial apoptosis, oxidative stress, inflammation, and fibrosis *in vivo* and *in vitro* by the mechanism of reduction of NF-κB p65 activation.

## Graphical Abstract

Gal-3 triggers myocardial apoptosis, oxidative stress, inflammatory cytokines release, macrophage infiltration, and fibrosis, leading to cardiac dysfunction in DCM mice. 
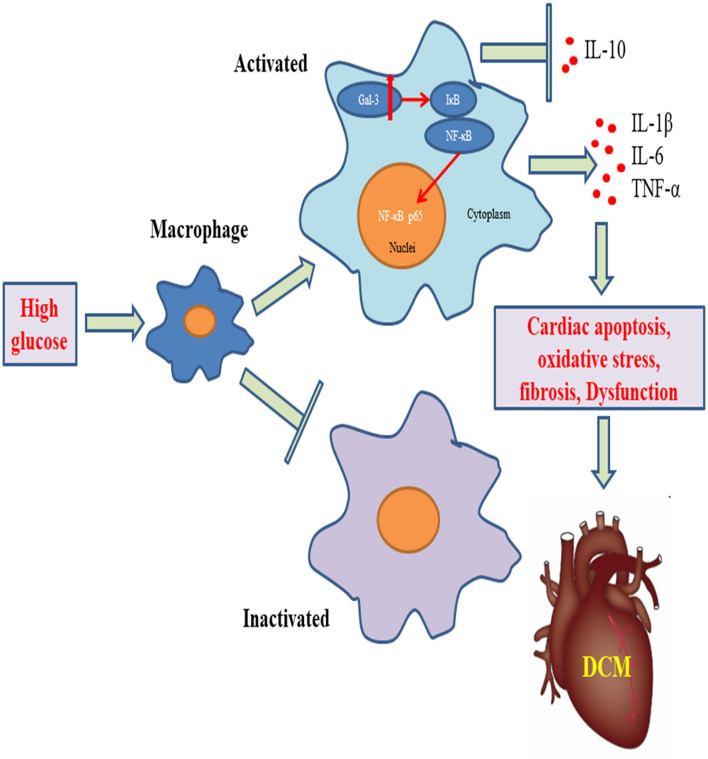

## Introduction

Type 2 diabetes mellitus (T2DM) is a global epidemic and is expected to affect over 693 million people worldwide by 2045 (1). Diabetic cardiomyopathy (DCM) was defined as a pathophysiological condition, in which heart failure (HF) occurred in the absence of coronary artery disease, hypertension, and valvular heart disease (2). Multiple pathophysiological factors in diabetes promote the development of cardiomyopathy from the early stages of diastolic cardiac fibrosis and stiffness/relaxation dysfunction to a later stage of systolic HF (3). The HF results in worsened clinical outcomes in patients with diabetes mellitus (4). Many commonly used antihyperglycemic therapies have successfully reduced hyperglycemia in diabetes, but these drugs have not reduced the high occurrence of HF (5). In addition, the protective effects of novel hypoglycemic drugs, such as sodium-glucose cotransporter 2 inhibitors (6, 7) and glucagon-like peptide-1 analogs (8), on HF are independent of the hypoglycemic effect. Furthermore, dapagliflozin has been shown to reduce the risk of HF worsening, regardless of diabetes status (9). As a result, factors other than glycemia may contribute to the risk of HF in patients with diabetes.

Many pathophysiological mechanisms, such as inflammation, oxidative stress, endoplasmic reticulum stress, aberrant insulin signaling, autophagy, myocardial metabolism, mitochondrial bioenergetics, and lipotoxicity, might be amenable to pharmacological therapy to decrease the risk of cardiac dysfunction in DCM. The role of maladaptive inflammation cytokines in the pathogenesis of DCM and HF has been established. Inflammatory cell infiltration, such as macrophages, may play a role in myocardial fibroblast collagen expression (10). Infiltrating macrophages and associated inflammatory cytokines can be implicated in diabetes-induced cardiac fibrosis and dysfunction (1). The activation of nuclear factor k-light-chain-enhancer of activated B cells (NF-kB) plays an important role in triggering cytokine expression and proinflammatory response (11). Furthermore, pro-inflammatory factors, such as NLRP3 inflammasome and the Toll-like receptor-4, promote DCM by the modulation of NF-kB (12, 13). Increased circulating levels of proinflammatory cytokines, such as tumor necrosis factor-α (TNF-α), interleukins (IL) 1 and 6, transforming growth factor-β (TGF-β), and monocyte chemotactic protein 1, also drive cardiac remodeling and fibrosis, and final diastolic dysfunction (14, 15). As a result, reducing inflammation macrophage infiltration is a promising strategy for DCM treatment.

Galectin-3 (Gal-3), which is a 30-kDa lectin secreted mainly by macrophages, contains a carbohydrate-recognition binding domain that binds to β-galactoside (16). Gal-3 plays an important disease-exacerbating role in autoimmune/inflammatory and malignant diseases (17–19). Gal-3 suppression attenuates many fibrotic diseases (20). In particular, Gal-3 plays a key role in cardiac fibrosis and remodeling. Cardiovascular fibroblast proliferation, collagen deposition, and ventricular dysfunction are all caused by recombinant Gal-3 (21). In a long-term transverse aortic constriction mouse model, inhibiting Gal-3 slows the progression of cardiac remodeling (22). Gal3 can also link inflammation to decreased insulin sensitivity (23). However, the role of Gal-3 in the progression of DCM and high glucose (HG)-induced macrophage activation remains unknown. In this study, we investigated whether Gal-3 contributes to the development of DCM *in vivo* and molecular mechanisms *in vitro*.

## Methods

### Animal Preparations and Experiments

All the animal procedures were approved by the Wenzhou Medical University Animal Policy and Welfare Committee and conformed to the National Institutes of Health Guidelines. Male C57BL/6 mice (18–22 g) were purchased from the Experimental Animal Center of Zhejiang Province (Hangzhou, Zhejiang, China). All animals were housed at a constant room temperature with a 12:12-h light-dark cycle and had free access to diet and water. The mice, aged 8 weeks old, received streptozotocin (STZ, 50 mg/kg) or vehicle (citrate buffer) by i.p. injection for 5 consecutive days. One week later, their fasting blood glucose levels were measured with a glucometer, and mice with glucose levels greater than 16.7 mmol/l were considered diabetic. The diabetic mice were randomly divided into four groups: a control group and diabetic mice received 2.5 × 10^10^ viral genomes of adeno-associated virus 9 (AAV-9) incorporating Gal-3-short hairpin RNA (shRNA) (AAV9-Gal-3) or an equal amount of AAV-9 incorporating scrambled-shRNA (AAV9-NC) *via* the tail vein injection, respectively. All mice were fed for another 16 weeks, and their blood glucose levels were measured weekly. Before detection, mouse hearts were isolated at 16 weeks and stored at −80°C or fixed in 4% paraformaldehyde.

### ELISA Analyses

The blood of mice was collected by retrobulbar bleeding and was centrifuged for 2,000 rpm for 20 min. The mice serum was stored at −80°C for further analyses. The concentrations of serum creatine kinase MB isoenzyme (CK-MB) and lactate dehydrogenase (LDH) were measured using commercial ELISA kits (Cloud-Clone, China). Heart issues or culture supernatants of RAW 264.7 macrophage cells were collected. The concentrations of IL-10, IL-1, IL-6, TNF-α, and TGF-β1 were also detected using commercial ELISA kits (Beyotime and DAKEWE, China).

### Echocardiography Analyses

The cardiac systolic and diastolic functions were determined using non-invasive transthoracic echocardiography in anesthetized mice (VEVO3100, Fujifilm VisualSonics). Left ventricular end-systolic diameter and end-diastolic diameter (LVESD, LVEDD), ejection fraction (EF), and fractional shortening (FS) were measured.

### Histological and Fibrotic Analyses

The paraffin-embedded cardiac tissues of mice were continuously sectioned at a thickness of 4 μm. Hematoxylin and eosin (HE) and Masson staining were performed according to the manufacturer's instructions. The morphology and fibrosis of heart tissue were examined under a light microscope (Nikon) at a magnification of 400 ×.

### TUNEL Staining

TUNEL staining was performed to detect cell apoptosis in the heart. After dewaxing and rehydrating, heart tissue sections (4 μm thick) were washed by phosphate-buffered saline (PBS) and permeabilized with Proteinase K (0.02 μg/μl). These sections were incubated with a TUNEL staining solution based on the manufacturer's recommendation. The percentage of TUNEL-positive cells was evaluated by a fluorescence microscope.

### Real-Time Quantitative PCR

Trizol was used to lyse heart tissues after they were harvested. The Primescript RT reagent kit was used for both reverse transcription and quantitative PCR (qPCR) (Takara, Japan). The light cycler 480 SYBR Green I Master (Roche, Switzerland) was used for qPCR analyses. The primer sequences of types I and III collagen (COL1A2 and COL3A1) were generated by our laboratory and are as follows: *COL1A2*, forward 5′-CACCCCAGCGAAGAACTCAT-3′, reverse 5′-TCTCCTCATCCAGGTACGCA-3′; *COL3A1A1*, forward 5′-GAGGAATGGGTGGCTATCCG-3′, reverse 5′-TCGTCCAGGTCTTCCTGACT-3′; and *GAPDH*, forward 5′-ATGGGTGTGAACCACGAGAA-3′, reverse 5′-ATGAGCCCTTCCACAATGCC-3′. The amount of each gene was detected and normalized to the amount of GAPDH.

### Immunofluorescence Analyses

Heart issues or RAW 264.7 macrophage cells were fixed in 4% paraformaldehyde for 15 min, permeabilized with 0.5% Triton X-100 for 10 min and incubated with blocking buffer for 30 min at room temperature. Specimens were then incubated overnight at 4°C with primary antibodies (CD68, Abcam, 1:200; Gal-3, Abcam, 1:200; eNOS, Abcam, 1:200) and 1 h at room temperature with secondary antibody. Cell nuclei were stained with DAPI and the signals were measured using a confocal laser microscope (Olympus, Japan).

### Western Blotting

Heart issues or RAW 264.7 macrophage cells were lysed with a Radioimmunoprecipitation assay (RIPA) buffer containing protease and phosphatase inhibitors. For nuclear and cytoplasmic protein analyses, the Nuclear and Cytoplasmic Protein Extraction Kit (Beyotime, Jiangsu, China) was used. Total protein concentrations were determined with the BCA protein assay kit (Beyotime). Protein samples (50 μg) were subjected to electrophoresis by 8–12% sodium dodecyl sulfate-polyacrylamide gel electrophoresis (SDS-PAGE), transferred to polyvinylidene fluoride (PVDF) membranes, and blocked in Tris-buffered saline containing 0.05% tween and 5% non-fat dry milk. Primary antibody (Gal-3, Abcam, 1:5,000; p-IκB, Santa Cruz, 1:500; IκB, Proteintech, 1:1,000; p-NF-κB p65, AFFINITY, 1:500; p50, Proteintech, 1:200; b-actin Atagenix, 1:3,000; GAPDH, Proteintech, 1:5,000) incubations were performed at 4°C overnight, and secondary antibodies were incubated for 1 h at room temperature. The immunoreactive bands were visualized using Enhanced Luminol Reagent and Oxidizing Reagent. Densitometric analyses were conducted using Quantity One software (Bio-Rad, Hercules, CA, USA).

### Statistical Analyses

The experimental data were expressed as the mean ± standard deviation (SD). The differences between groups were analyzed with Student's *t*-test or one-way analysis of variance using GraphPad Pro5.0 (GraphPad, San Diego, CA, USA). All *p* < 0.05 were considered statistically significant.

## Results

### Gal-3 Inhibition Decreased Diabetes-Induced Cardiac Injury

**Figure 1:**
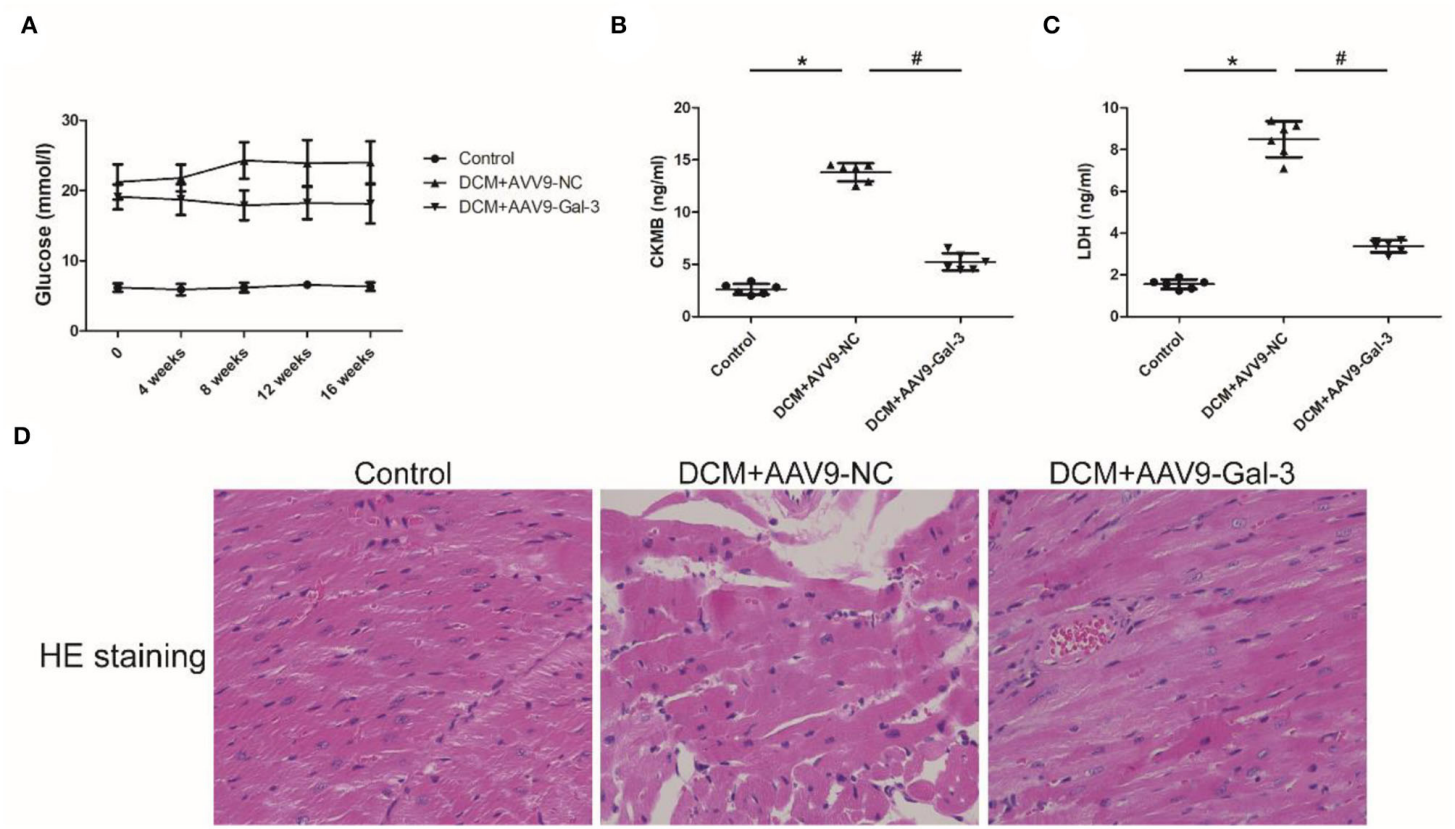
Gal-3 inhibition decreased diabetes-induced cardiac injury **(A)** blood glucose level was measured during 16 weeks after STZ injection. **(B,C)** ELISA analysis of heart CK-MB and LDH expression at 16 weeks after STZ injection. **(D)** HE staining of heart tissue at 16 weeks after STZ injection. Magnification, 200 ×. Data are shown as mean ± SD. n = 6, ^*^P < 0.05 vs. Control; ^#^ *P*<*0.0*5 vs. DCM+ AAV9-NC.

Streptozotocin (STZ) caused an increase in the blood glucose level in the DCM group, and the blood glucose level was maintained during the experiment interval (Figure 1A). However, the blood glucose level was significantly decreased in the DCM+AAV9-Gal-3 group. Furthermore, the ELISA assay revealed that myocardial injury markers (CK-MB and LDH) were upregulated in the DCM group, whereas Gal-3 inhibition reduced the increase (Figures 1B,C). The myocardial structure was examined by HE-staining. Diabetes induced histological abnormalities, whereas treatment with AAV9-Gal-3 markedly reversed these abnormalities (Figure 1D).

### Gal-3 Inhibition Reversed Diabetes-Induced Cardiac Dysfunction

**Figure 2:**
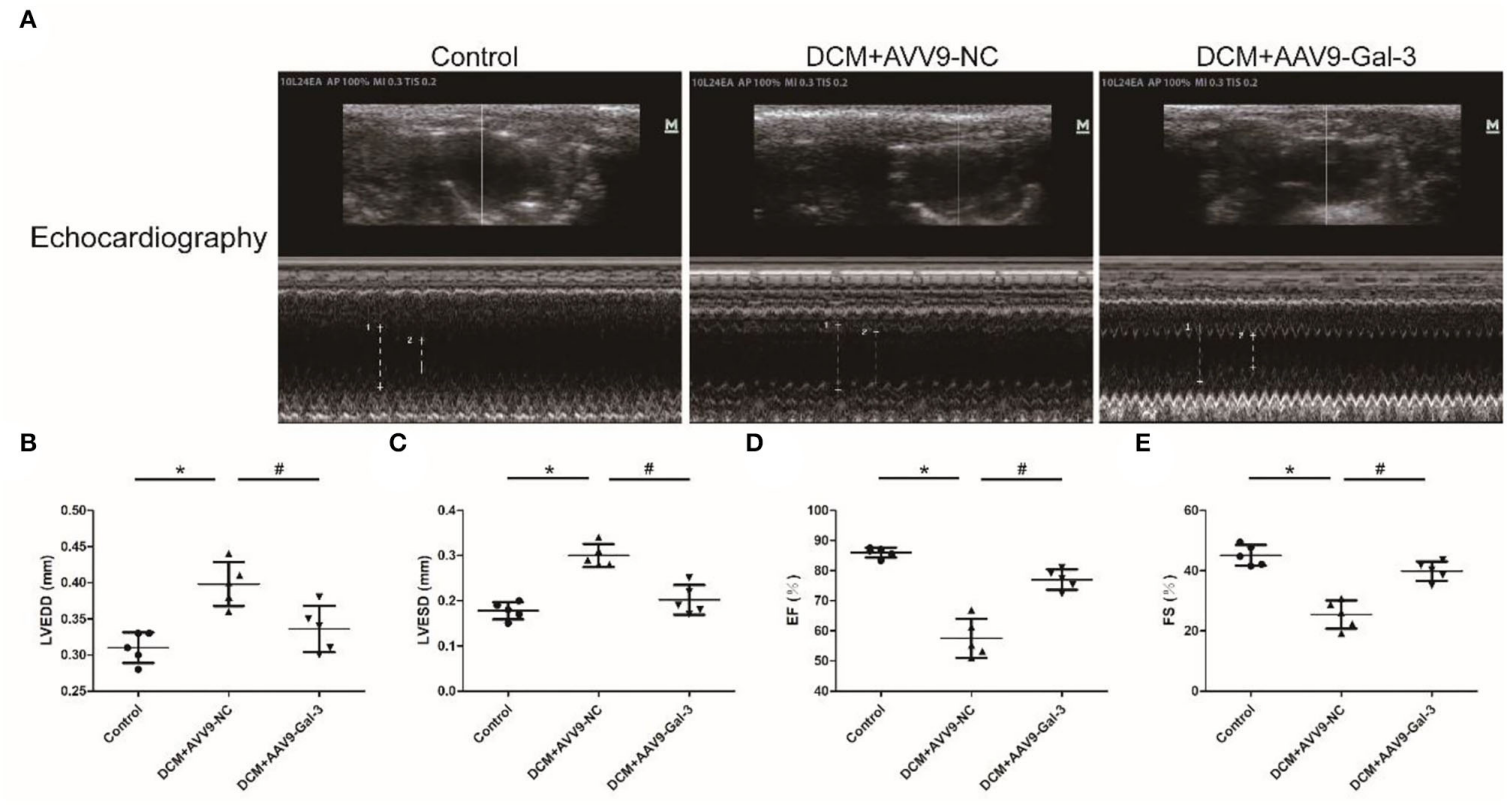
Gal-3 inhibition reversed diabetes-induced cardiac dysfunction **(A–E)** echocardiographic measurement of cardiac structure and function. Data are shown as mean ± SD. *n* = 5, *P < 0.05 vs. Control; ^#^ *P* < 0.05 vs. DCM+ AAV9-NC.

Cardiac structure and function were examined by echocardiographic measurement (Figure 2A). Diabetic mice had cardiac systolic and diastolic dysfunction, as evidenced by increased LVEDD and LVESD (Figures 2B,C), as well as decreased EF% and FS% (Figures 2D,E). Improved cardiac systolic and diastolic dysfunction was also observed by Gal-3 inhibition.

### Gal-3 Inhibition Attenuated Diabetes-Induced Cardiac Fibrosis

**Figure 3:**
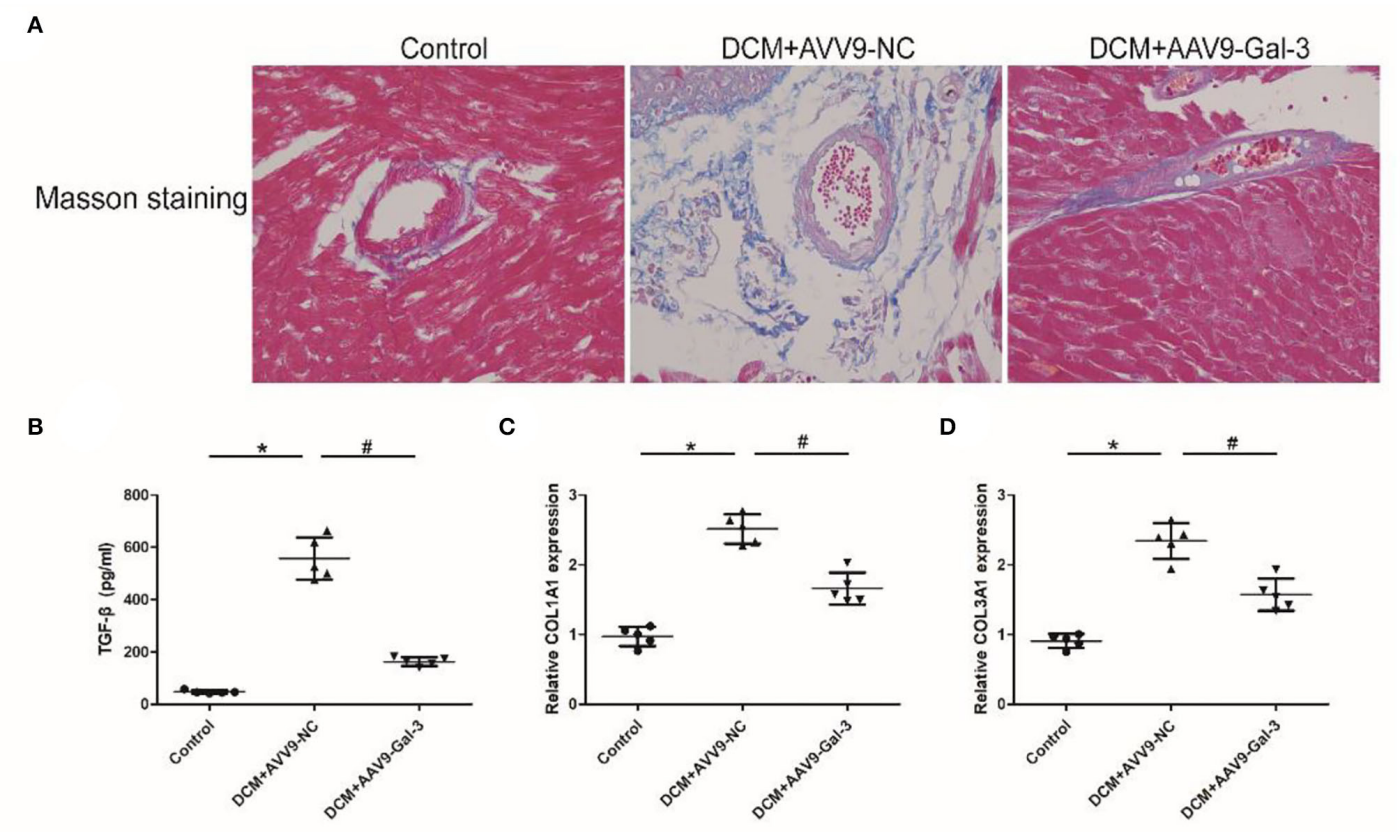
Gal-3 inhibition attenuated diabetes-induced cardiac fibrosis **(A)** Masson staining of heart tissue at 16 weeks after STZ injection. Magnification, 200 ×. **(B)** ELISA analysis of heart TGF-β expression. **(C,D)** Real-time PCR analysis of COL1A2 and COL3A1 mRAN level. Data are shown as mean ± SD. *n* = 5, *P < 0.05 vs. Control; ^#^ *P* < 0.05 vs. DCM+ AAV9-NC.

Masson staining showed Gal-3 inhibition ameliorated myocardial fibrosis in diabetic mice (Figure 3A). The ELISA and real-time PCR analyses revealed a significant increase in profibrotic makers, including TGF-β (Figure 3B) and COL1A2 and COL3A1 (Figures 3C,D) expressions in diabetic hearts.

### Gal-3 Inhibition Decreased Diabetes-Induced Apoptosis and Oxidative Stress in the Heart

**Figure 4:**
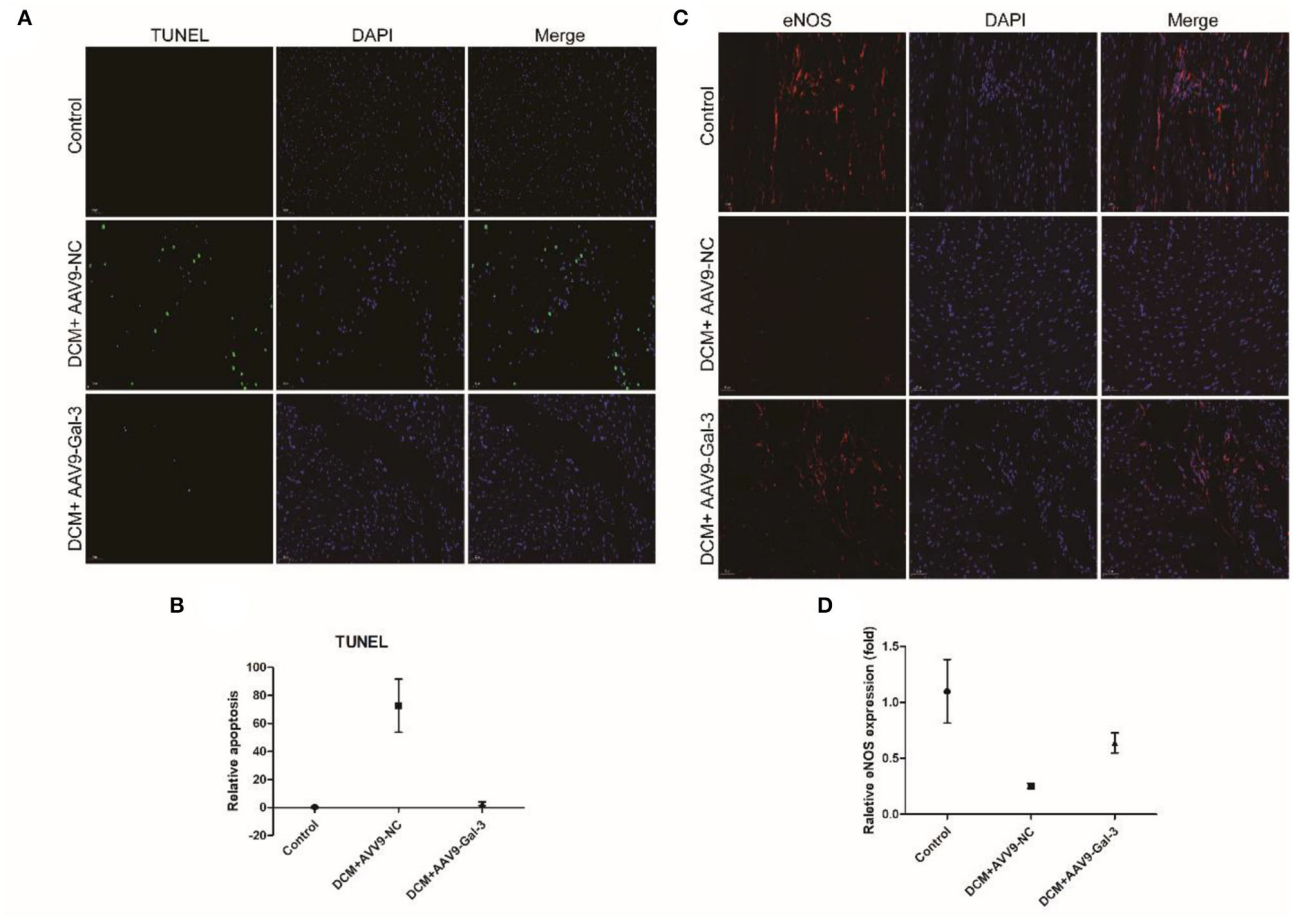
Gal-3 inhibition reduced diabetes-induced apoptosis and oxidative stress **(A,B)** TUNEL and **(C,D)** immunofluorescence staining of eNOS in heart tissue at 16 weeks after STZ injection. Data are shown as mean ± SD. *n* = 3, *P < 0.05 vs. Control; ^#^*P* < 0.05 vs. DCM+ AAV9-NC.

The TUNEL staining showed diabetes-induced cardiac apoptosis, while Gal-3 inhibition reduced apoptosis (Figures 4A,B). Diabetes-induced downregulations of antioxidant marker-endothelial nitric oxide synthase (eNOS) were reversed by Gal-3 inhibition (Figures 4C,D).

### Gal-3 Inhibition Modulated Diabetes-Induced Cytokine Expression

**Figure 5:**
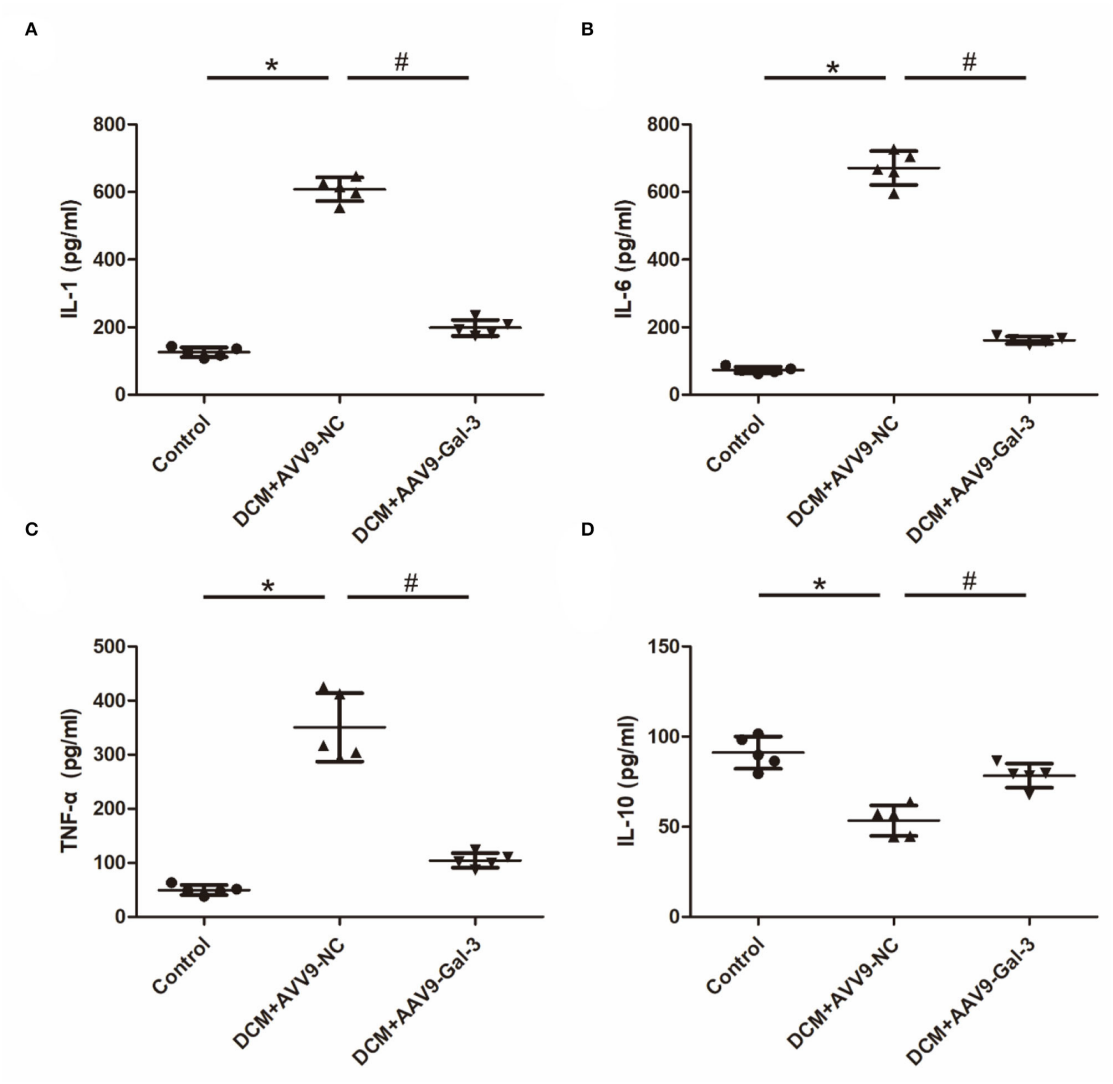
Gal-3 inhibition modulated diabetes-induced cytokines expression **(A–D)** ELISA analysis of IL-1, IL-6, TNF-α and IL-10. Data are shown as mean ± SD. *n* = 5, *P < 0.05 vs. Control; ^#^*P* < 0.05 vs. DCM+ AAV9-NC.

The increase of proinflammatory cytokines, including IL-1, IL-6, and TNF-α (Figures 5A–C), and the decrease of anti-inflammatory cytokines (IL-10) (Figure 5D) were observed in the DCM group. However, the decrease of IL-1, IL-6, and TNF-α and the increase of IL-10 were observed in the DCM+AAV9-Gal-3 group.

### Gal-3 Inhibition Reduced Diabetes-Induced Macrophage Infiltration and NF-κB p65 Activation

**Figure 6:**
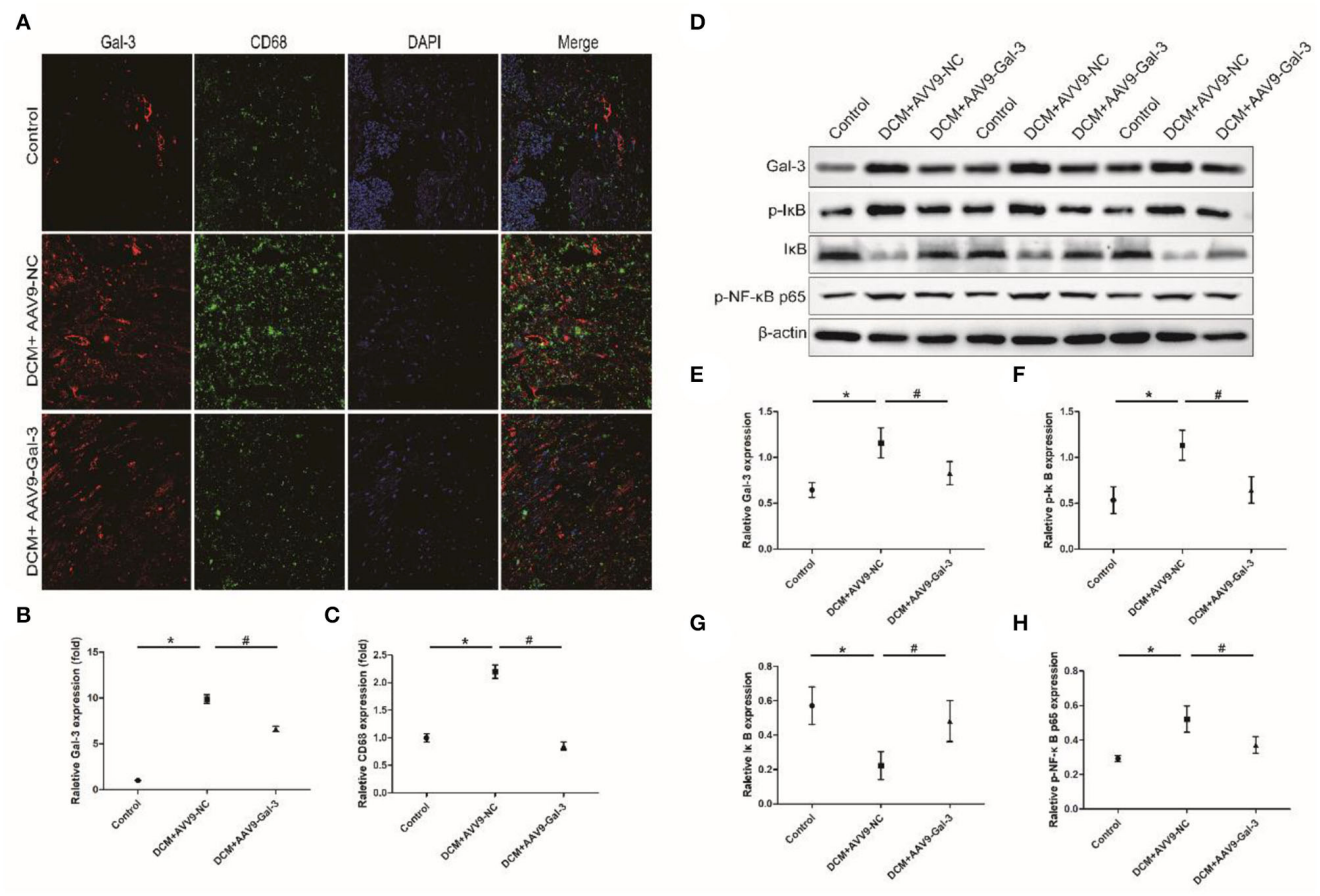
Gal-3 inhibition reduced diabetes-induced macrophage infiltration and NF-κB p65 activation. **(A–C)** Immunofluorescence staining of CD68 and Gal-3 in heart tissue at 16 weeks after STZ injection. **(D–H)** Western bloting analysis of Gal-3, p-IκB, IκB and p- NF-κB p65. Data are shown as mean ± SD. *n* = 3, *P < 0.05 vs. Control; ^#^ *P* < 0.05 vs. DCM+ AAV9-NC.

Immunofluorescence staining indicated that macrophage marker (CD68) was significantly upregulated induced by STZ, whereas Gal-3 inhibition reduced CD68 expression (Figures 6A–C). Western blot analyses revealed that p-IκB and p-NF-κB p65 were increased, and IκB was decreased by diabetes, which was both reversed by Gal-3 inhibition (Figures 6D–H).

### Gal-3 Knockdown Regulated HG-Induced Cytokine Expression in Macrophages

**Figure 7:**
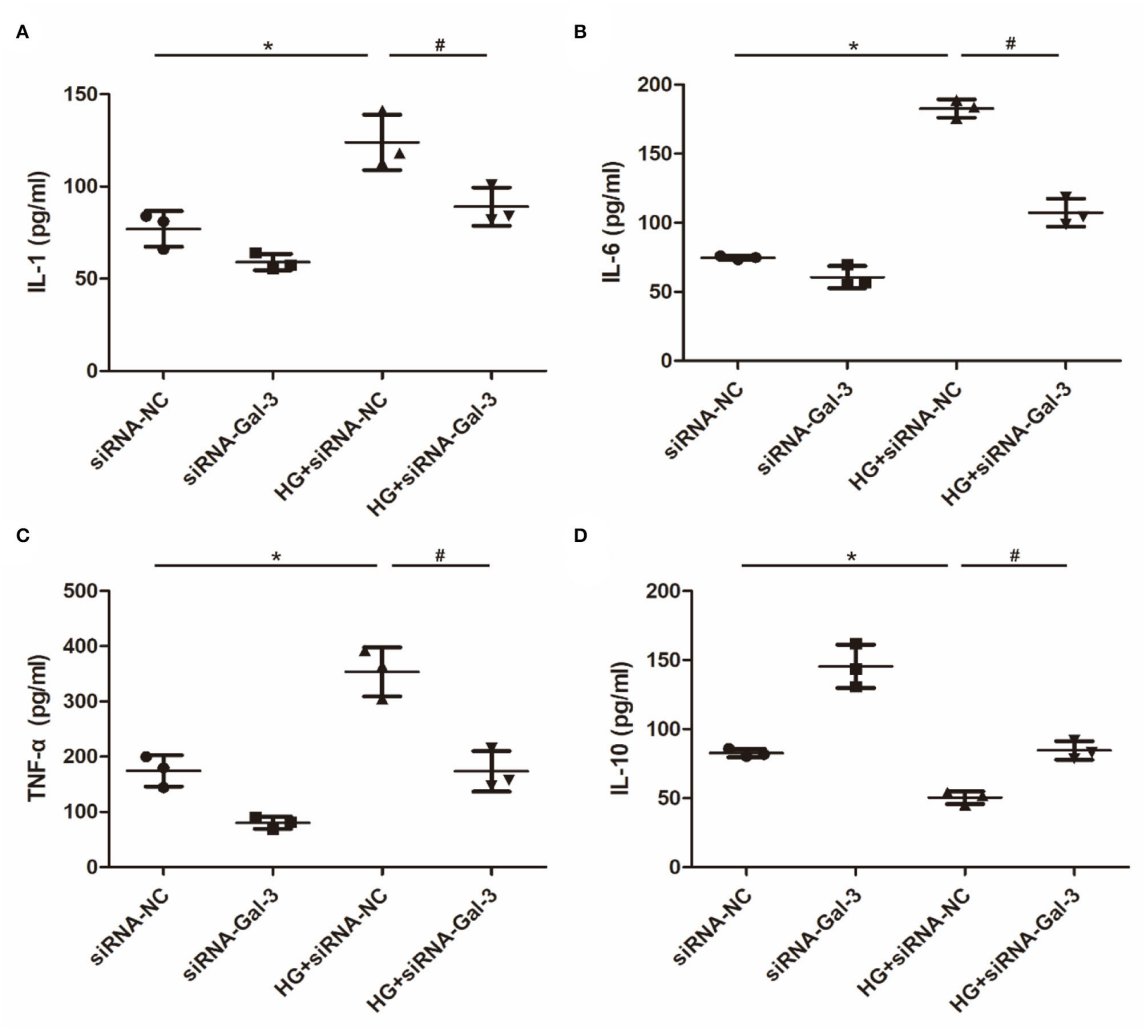
Gal-3 knockdown regulated HG (high glucose)-induced cytokines expression in macrophages **(A–D)** ELISA analysis of IL-1, IL-6, TNF-α and IL-10. Data are shown as mean ± SD. *n* = 3, *P < 0.05 vs. siRNA-NC; ^#^*P* < 0.05 vs. HG+ siRNA-NC.

Proinflammatory cytokines, including IL-1, IL-6, and TNF-α (Figures 7A–C), were increased and anti-inflammatory cytokines (IL-10) were decreased by HG (Figure 7D). However, Gal-3 knockdown reversed the increase of IL-1, IL-6, and TNF-α and the decrease of IL-10.

### Gal-3 Knockdown Induced HG-Induced Macrophage Infiltration

**Figure 8:**
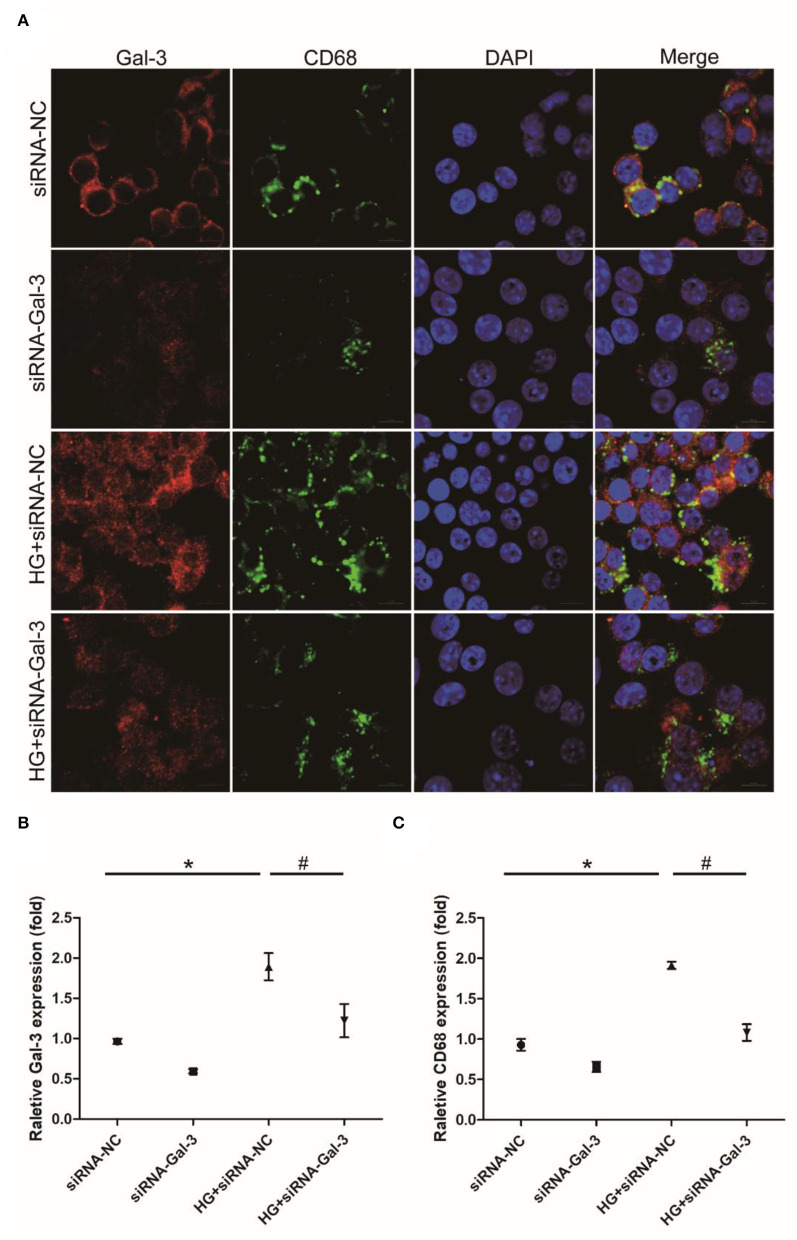
Gal-3 knockdown induced HG-induced macrophage infiltration **(A)** Immunohistochemical staining of CD68 and Gal-3. Representative analysis of CD68 **(B)** and Gal-3 **(C)**. Data are shown as mean ± SD. *n* = 3, *P < 0.05 vs. siRNA-NC; ^#^ *P* < 0.05 vs. HG+ siRNA-NC.

Immunofluorescence staining showed that CD68 was increased by HG, which was attenuated by Gal-3 knockdown (Figures 8A–C).

### Gal-3 Knockdown Reduced NF-κB p65 Activation

**Figure 9:**
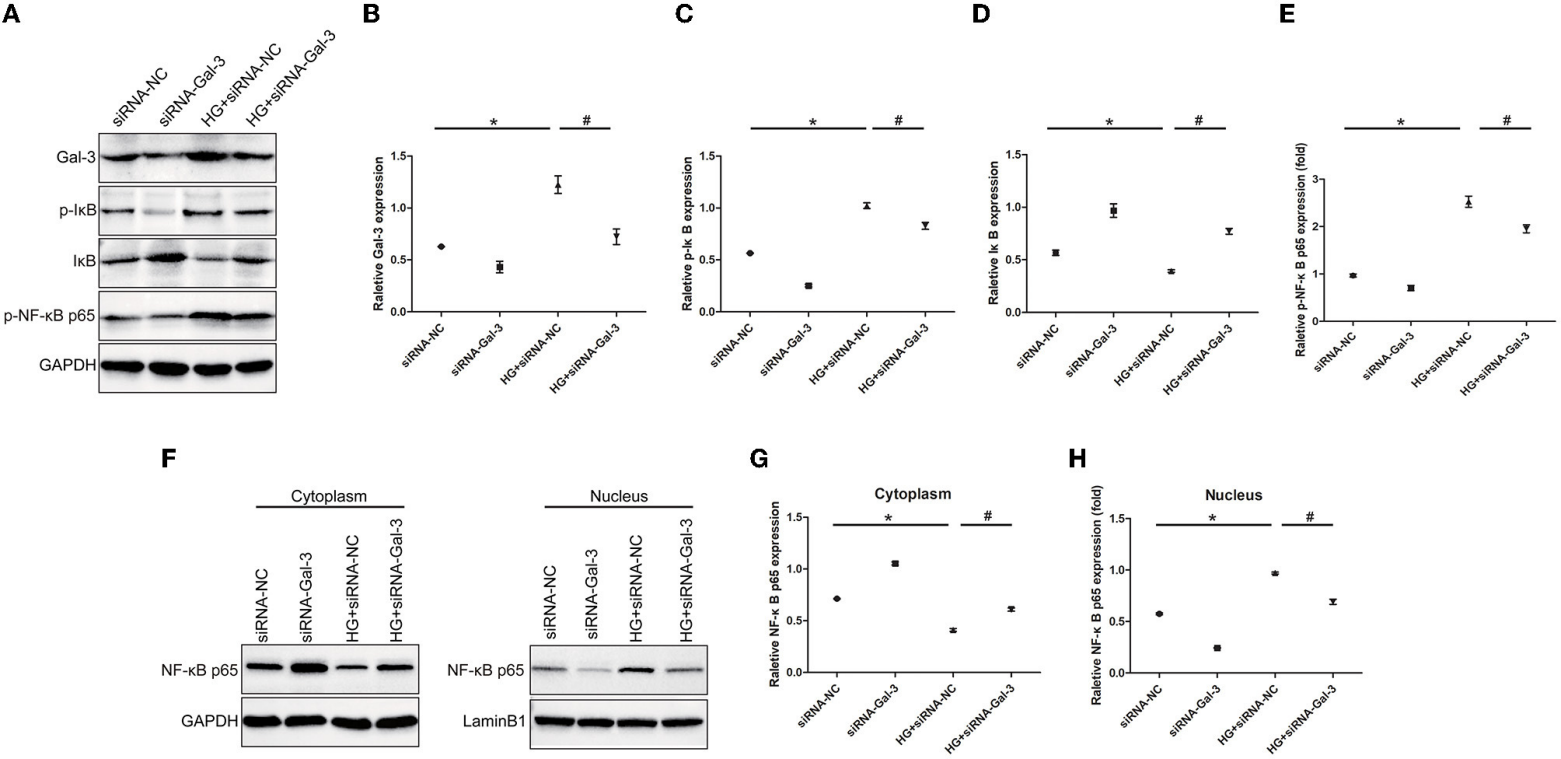
Gal-3 knockdown reduced NF-κB p65 activation Western bloting analysis of Gal-3, p-IκB, IκB and p- NF-κB p65 **(A)**. Representative analysis of Gal-3 **(B)**, p-IκB **(C)**, IκB **(D)** and p-NF-kB p65 **(E)**. **(F–H)** Western blotting analysis of NF-κB p65 in cytoplasm and nucleus. Representative analysis of NF-κB p65 in cytoplasm and nucleus. Data are shown as mean ± SD. *n* = 3, *P < 0.05 vs. siRNA-NC; ^#^ *P* < 0.05 vs. HG+ siRNA-NC.

Western blot analyses showed that HG-induced p-IκB and p-NF-κB p65 expression and decreased IκB (Figures 9A–E). The Gal-3 knockdown reduced p-IκB and p-NF-κB p65, as well as increased IκB. Furthermore, NF-κB p65 was decreased in the cytoplasm but increased in the nucleus (Figures 9F–H). Gal-3 knockdown reduced NF-κB p65 in the nucleus and increased it in the cytoplasm.

## Discussion

Our study results suggested that inhibition of Gal-3 alleviated cardiac injury and myocardial apoptosis, oxidative stress, and fibrosis in STZ-induced DCM. In addition, inhibiting Gal-3 knockdown resulted in the suppression of proinflammatory cytokines and macrophage infiltration *in vivo via* the mechanism of NF-κB p65 inactivation. *In vitro*, Gal-3 knockdown also blunted HG-induced inflammatory cytokines and macrophage infiltration, as well as NF-κB p65 inactivation. Our data identified that Gal-3 regulates DCM by the blockage of inflammation and NF-κB p65 activation. Hence, targeting Gal-3 may be a promising strategy for DCM treatment.

The DCM is caused by a complex set of pathophysiological factors. Cardiac fibrosis, which is caused by these abnormalities, is a major contributor to stiffness/diastolic dysfunction and, later, systolic dysfunction (3). The Gal-3 level is related to markers of the cardiac extracellular matrix and, therefore, emerges as a biomarker associated with death or HF hospitalization (24). The use of Gal-3 for prognosis in patients with moderate to severe HF was recommended in the 2013 American College of Cardiology Foundation/American Heart Association guidelines for the management of HF (25). New research has highlighted the use of Gal-3 as a drug target. We discovered that DCM treated with Gal-3 knockdown at the start of the experiment improved cardiac function and reduced cardiac injury biomarkers. Clinical studies have also revealed that plasma Gal-3 is an independent predictor of HF outcomes and myocardial function in patients with preserved EF, but not in patients with reduced EF (24, 26, 27). In this study, in the stage of systolic dysfunction, Gal-3 remains markedly high, indicating that further studies should be conducted to identify the role of Gal-3 in the stage of systolic dysfunction. Furthermore, Gal-3 inhibition was found to reduce fibrosis and profibrotic markers in the DCM model, including TGF-β, COL1A2, and COL3A1.

Emerging evidence indicates that Gal-3 regulates cardiac fibrosis *via* inflammation. The pharmacological inhibition of Gal-3 prevented cardiac dysfunction and fibrosis (28). Gal-3 blockade inhibited proinflammatory and profibrotic markers, including chemokine ligand 2 (CCL2), TNF-α, and IL-1β in human cardiac fibroblasts (29). In addition, Gal-3 blockage could also inhibit isoproterenol-induced cardiac inflammation and fibrosis (30). Gal-3 blockage decreased obesity-induced cardiac dysfunction, inflammatory markers osteopontin, and CCL2, and fibrosis markers' collagen type I, TGF-β, and connective tissue growth factor (31). Gal-3 stimulated a variety of profibrotic factors, especially the phagocytosis of apoptotic cells and the production of cellular debris from macrophages (32). Increased Gal-3 expression in macrophages causes alternative macrophage activation and promotes cardiac remodeling after myocardial infarction (33). Gal-3 in CD206+ macrophages has also been reported to result in reparative fibrosis in myocardial infarction (34). A previous study demonstrated that CD11b-F4/80^++^ macrophage infiltration at 4 weeks STZ-induced diabetes was increased, but cardiac inflammation resolved at 12 weeks (35). CD68 has been used more frequently as a macrophage marker in the heart (36, 37) and CD11b+ as a macrophage marker in the liver and skin (38). More importantly, in the previous study, CD68 was not detected. We discovered that another macrophage maker, CD68, was still increased after 16 weeks. Furthermore, the STZ challenge increased proinflammatory cytokine profiles, while reducing anti-inflammatory cytokine profiles. Systemic inflammation, including circulating cytokines, chemokines, immune cells, and other inflammatory biomarkers, is present in both types of patients with diabetes (39, 40). The induction of proinflammatory cytokines (TNF-α, IL-1β, and IL-6) has also been identified after the increase of systemic inflammatory markers in the heart of diabetic models (41–43). It has been reported that IL-10 is upregulated in reparative and pressure overload-induced fibrosis and that it is localized in T lymphocytes and macrophages infiltrating the remodeling heart. The IL-10 may regulate the cardiac fibrotic response in addition to its well-documented anti-inflammatory properties (44, 45). Though the conflict between its pro-and antifibrotic actions remains (46–48), IL-10 has been shown to have anti-inflammatory and antifibrotic properties in STZ-induced DCM (49, 50). Our data were consistent with these studies.

The HG-induced IL-1β release in human macrophages (51). In RAW264.7 macrophages, HG significantly increased the mRNA level, as well as the release of IL-1 and TNF-α (52–54). Though the role of Gal-3 in macrophage activation and proinflammation has been studied, its involvement in HG-induced inflammatory cytokine release has not been identified. In RAW264.7 macrophages, we discovered that HG induced the release of IL-1β, IL-6, and TNF-α, which was inhibited by Gal-3 inhibition.

The NF-κB is a heterodimer made up of the p50 and RelA/p65 subunits that act as a proinflammatory transcription factor and mediates the inflammatory response in macrophages (55). Under unstimulated conditions, NF-κB is localized in the cytoplasm binding IκB (56). The IκB phosphorylation and downregulation leave the NF-κB dimer free to translocate to the nucleus and initiate the transcription of targeting genes, including IL-1β and TNF-α (57). When cells are stimulated by environmental factors, NF-κB is released, allowing it to translocate to the nucleus, where it continues to mediate the transcription of target genes, such as IL-1β and TNF-α.

In macrophages, NF-κB p65 phosphorylation and localization in the nucleus also mediated inflammatory cytokines (58–60), which was correlated to many diseases (61–63). Furthermore, NF-κB p65 activates macrophage infiltration, inflammation, and myocardial fibrosis in DCM (64, 65). The HG induced NF-κB activation in RAW264.7 macrophages (66). The Gal-3 inhibitor modified citrus pectin has been proven to downregulate the expression of Gal-3 and NF-κB-p65 activation (67). In this study, we first discovered that Gal-3 inhibition blocked NF-κB-p65 activation *in vivo*. We also identified that Gal-3 inhibition modulates HG-induced NF-κB-p65 phosphorylation and nuclear translocation. Therefore, the Gal-3/NF-κB-p65 regulatory network provides novel insights into the pathogenesis and treatment of DCM.

## Conclusion

In conclusion, our findings point to a mechanism by which Gal-3 may promote NF-κB-p65 activation, thereby alleviating DCM. The AAV9 intervention may provide a new therapeutic strategy for DCM and related heart diseases because of the potential role and therapeutic value of Gal-3 in fibrotic heart diseases.

## Limitation

There are some limitations to the present study. Firstly, we failed to identify a novel mechanism of Gal-3 modulating DCM. Secondly, it is insufficient to determine that Gal-3 inhibition leads to ameliorating DCM by NF-κB-p65 activation. More experiments should be performed in the future.

## Data Availability Statement

The original contributions presented in the study are included in the article/supplementary material, further inquiries can be directed to the corresponding author/s.

## Ethics Statement

The animal study was reviewed and approved by Wenzhou Medical University Animal Policy and Welfare Committee.

## Author Contributions

NZ designed, drafted, and revised the manuscript. LZ and XZ performed the research and wrote the manuscript. BH and WX collected and analyzed the data. All the authors read and approved the final manuscript.

## Funding

This research was supported by the Zhejiang Provincial Natural Science Foundation of China under Grant No. LQ20H020011.

## Conflict of Interest

The authors declare that the research was conducted in the absence of any commercial or financial relationships that could be construed as a potential conflict of interest.

## Publisher's Note

All claims expressed in this article are solely those of the authors and do not necessarily represent those of their affiliated organizations, or those of the publisher, the editors and the reviewers. Any product that may be evaluated in this article, or claim that may be made by its manufacturer, is not guaranteed or endorsed by the publisher.

## Abbreviations

Gal-3: Galectin-3
DCM: Diabetic cardiomyopathy
i.p.: intraperitoneal injection
STZ: Streptozotocin
AAV-9: Adeno-associated virus 9
HF: Heart failure
HE: hematoxylin and eosin
CK-MB: creatine kinase MB isoenzyme
LDH: lactate dehydrogenase
COL1A2, Collagen, type I: alpha 1
COL3A1, Collagen, type III: alpha 1
LVEDD: Left ventricular end-diastolic diameter
LVEF, Left ventricular fractional ejection fraction, LVESD: Left ventricular end-systolic diameter
LVFS: Left ventricular fractional shortening fraction
TNF-α: tumor necrosis factor-α
TGF-β: transforming growth factor-β.
